# Optimization of Photosensitized Tryptophan Oxidation in the Presence of Dimegin-Polyvinylpyrrolidone-Chitosan Systems

**DOI:** 10.1038/s41598-018-26458-6

**Published:** 2018-05-23

**Authors:** Anna B. Solovieva, Valeria V. Kardumian, Nadezhda A. Aksenova, Lyudmila V. Belovolova, Mikhail V. Glushkov, Evgeny A. Bezrukov, Roman B. Sukhanov, Svetlana L. Kotova, Peter S. Timashev

**Affiliations:** 1 N.N. Semenov Institute of Chemical Physics, Department of Polymers and Composites, 4 Kosygin St., 119991 Moscow, Russia; 2 Institute for Regenerative Medicine, I. M. Sechenov First Moscow State Medical University, 8 Trubetskaya St., Moscow, 119991 Russia; 3 A.M. Prokhorov Institute of General Physics, 38 Vavilov St., 119991 Moscow, Russia; 4 Department of Urology, I. M. Sechenov First Moscow State Medical University, 8 Trubetskaya St., Moscow, 119991 Russia; 5 Institute of Photonic Technologies, Research center “Crystallography and Photonics”, 2 Pionerskaya St., Troitsk, Moscow, 142190 Russia

## Abstract

By the example of a model process of tryptophan photooxidation in the aqueous medium in the presence of a three-component photosensitizing complex (porphyrin photosensitizer-polyvinylpyrrolidone- chitosan, PPS-PVP-CT) in the temperature range of 20–40 °С, we have demonstrated a possibility of modification of such a process by selecting different molar ratios of the components in the reaction mixture. The actual objective of this selection is the formation of a certain PPS-PVP-CT composition in which PVP macromolecules would coordinate with PPS molecules and at the same time practically block the complex binding of PPS molecules with chitosan macromolecules. Such blocking allows utilization of the bactericidal properties of chitosan to a greater extent, since chitosan is known to depress the PPS photosensitizing activity in PPS-PVP-CT complexes when using those in photodynamic therapy (PDT). The optimal composition of photosensitizing complexes appears to be dependent on the temperature at which the PDT sessions are performed. We have analyzed the correlations of the effective rate constants of tryptophan photooxidation with the photophysical characteristics of the formed complexes.

## Introduction

Porphyrin photosensitizers (PPS) are macro heterocyclic pigments with peculiar physico-chemical properties which determine their extensive use in the medicine and catalysis. The presence of a system of conjugated double bonds and donor-acceptor side substituents results in the PPS propensity to intermolecular interactions in both solutions and condensed state leading to aggregation of macrocycles^1–5^. Many functional properties of aggregated macrocycles (spectral, photochemical, acidic-basic) are essentially altered, as compared to the molecular state of the same substances. In particular, aggregated porphyrins have a diminished photocatalytic and photodynamic PPS activity^6,7^. This effect is conventionally associated with the fact that aggregation of porphyrins and their analogues leads to non-radiative relaxation of the singlet excited ${P}_{2}^{\ast }$ state of the dye molecules and a decrease in the probability of their transition in the triplet excited ${P}_{3}^{\ast }$ state. It is the interaction of excited PPS molecules in the ${P}_{3}^{\ast }$ state with oxygen molecules that results in the generation of excited singlet oxygen molecules $({}^{1}O_{2}^{\ast })$. Therefore, the energy dissipation of the singlet excited state occurring due to aggregation of porphyrin molecules results in the decrease of the photosensitizing activity of porphyrin aggregates^8–10^.

The most pronounced reduction of the degree of macrocycle aggregation and increase of the PPS functional activity may be achieved by mixing the photosensitizers with surfactants, which are efficient dispersers preventing aggregation processes in porphyrin solutions^11^. Due to their ability to form micelles, surfactants may also solubilize water-insoluble porphyrin substances and transfer them into a water-soluble state suitable for their utilization in PDT. It should be pointed out that water-soluble porphyrins and their analogues – chlorins – may form complexes with surfactants, that boosts their photodynamic and photocatalytic activity^12^. In particular, appearance of weakly bound complexes of chlorine e6 in the form of a trisodium salt (photoditazine) with some amphiphilic polymers with surfactant properties (Pluronic® F127, polyvinylpyrrolidone, PVP) due to hydrophobic interactions was earlier detected by 1H NMR. Such a complex formation resulted in a decrease of the PPS aggregation degree and enhancement of their photocatalytic activity^13^.

The use of photoditazine complexes with PVP and Pluronic® F127 in the PDT of model wounds in rats in the early phase of the wound healing process has shown that amphiphilic polymers tend to reduce the PD-action toxicity. In particular, they are capable of preventing the development of a local hemorrhagic reaction, complicating the wound healing in the PDT, they also quench the inflammatory processes and boost the reparative processes in the wound^14^. Indeed, in the photoditazine –PVP experimental group in^14^ the amount of the exudate was lower than that in the group treated with photoditazine only. At the same time, the granulation tissue at the wound bed was thicker in the photoditazine –PVP group, the tissue was more mature and rich in vascular elements. The characteristic feature of this granulation tissue was also the presence of numerous proliferating fibroblasts and an increased number of macrophages, which indicated activation of the reparative processes. The signs of inflammation (oedema, neutrophil infiltration, erythrocyte stasis and sludge in capillaries) was less pronounced than it was in the photoditazine group.

The new tendencies in the improvement of the PDT efficacy in the treatment of hard-to-heal purulent wounds, trophic ulcers, infected burns, are exhibited in a combination of PDT with the effects of biologically active substances – enzymes, vitamins, natural bactericidal polymers, accelerating the healing process^15,16^. As one of such substances, chitosan (CT), a biologically active and bactericidal polysaccharide, is considered. However, the preliminary experience of its application as a polymer component in complex with PPS in model processes of photosensitized oxidation of organic substrates unambiguously indicated a diminished efficiency of PPS-CT systems. At the same time, as was found before^17^, some amphiphilic polymers with surfactants properties, in particular, PVP, in complex with PPS promoted a significant (by 3–5 times) boost of the photosensitizing activity of the photosensitizer drug forms used for PDT. Such an increase of activity was found to be related to the surfactant properties of PVP, which facilitated disaggregation of PPS associates, resulting in the enhancement of specific (per single photosensitizing complex) photocatalytic activity.

Taking into account all the mentioned findings, one has an opportunity to optimally select the ratio of components in a three-component PPS-PVP-CT photosensitizing complex, which exhibits high (at least, at the PPS level) photocatalytic activity. The objective of this study is to demonstrate such an opportunity by the example of a model (test) process of tryptophan oxidation in the aqueous medium in the temperature range characteristic for PDT sessions. In more detail, the temperature range involves those of the human body (36.6–36.9 °С) and those used in the PDT of experimental animals (38.5–39.5 °С). The actual task consists in creating such a composition of the PPS, PVP and CT components in the complex in which PVP molecules coordinate with the PPS molecules and at the same time virtually block formation of the complex bonds of the PPS molecules with CT molecules. A real solution may be found in an experimental study of the mentioned model process of tryptophan oxidation in the temperature range of 20–40 °С while selecting different molar ratios of the components in the reaction mixture. The known published data^18–21^ on the conformational lability of low- molecular-mass CT molecules in the mentioned temperature range provide a basis for such a search.

## Results and Discussion

As was shown earlier, photocatalytic activity of water-soluble porphyrins in the model process of tryptophan oxidation in aqueous solutions increased in the presence of amphiphilic polymers, in particular, polyethyleneglycol (M.W. 1.0 × 10^4^ Da), Pluronic F127 (M.W. 1.2 × 10^4^ Da) and polyvinylpyrrolidone (M.W. 2.0 × 10^4^ Da), while it dropped in the presence of chitosan (M.W. 3 × 10^6^ Da). Such a decrease of the PPS activity is related to the interaction of porphyrins with protonated aminogroups of the polysaccharide and the following aggregation of porphyrin molecules^22,23^.

**Figure 1:**
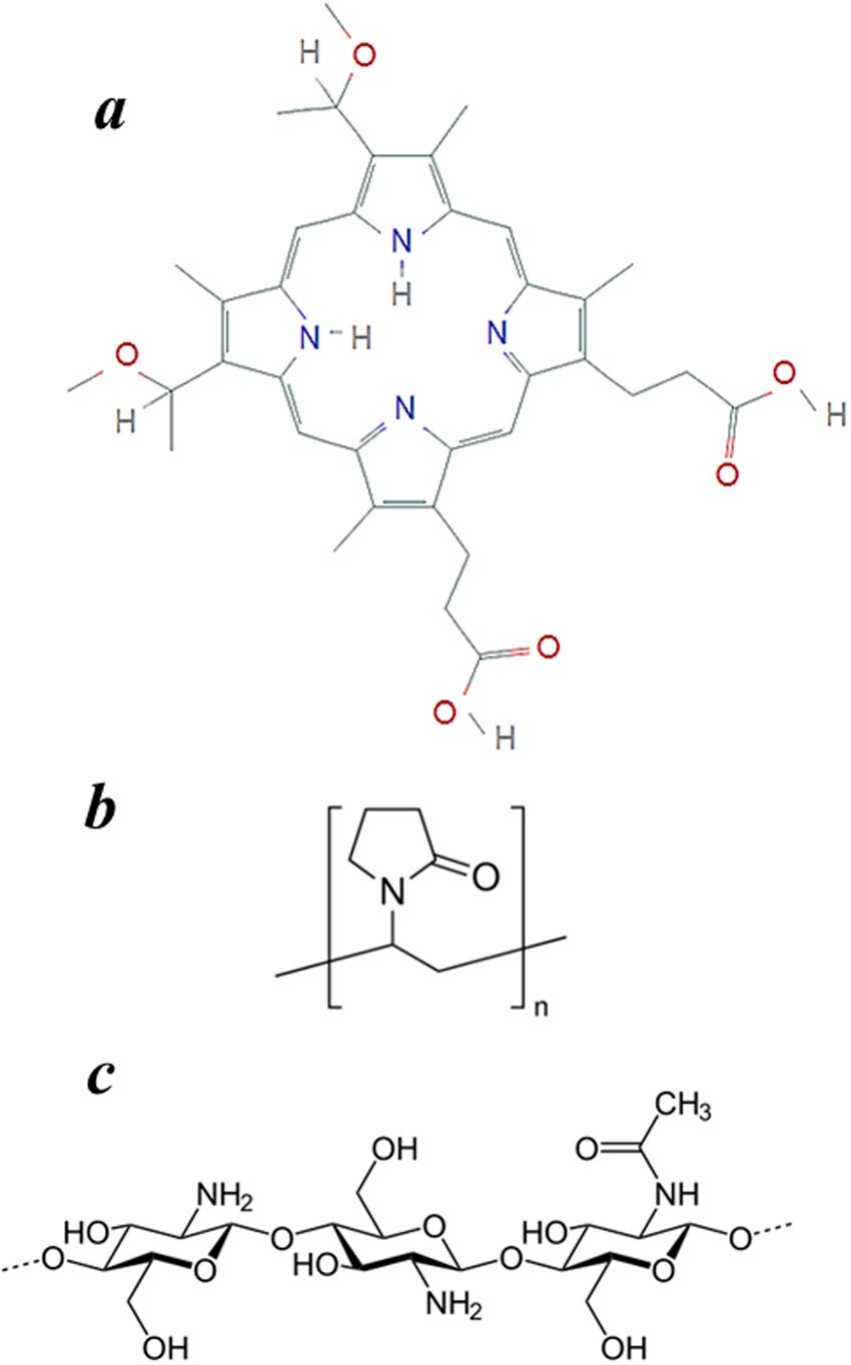
(**a**) Disodium salt of 3,8-di(1-methoxyethyl)deuteroporphyrin IX (DMG): (**b**) poly-N-vinylpyrrolidone (PVP): (**c**) chitosan (CT).

In this study, we used ternary systems based on dimegin (DMG) as a photosensitizer (Fig. 1а), PVP (Fig. 1b) as an amphiphilic polymer and chitosan (CT, Fig. 1c) as a biologically active polysaccharide. According to the data of our preliminary experiments for ternary DMG-PVP-CT photocatalytic systems used in PDT, the use of chitosan preparations with lower molecular weights is recommended. In this study, we used CT with M.W. 2.0 × 10^4^ Da. At the same time, as compared to the previous studies, the general character of variation of the specific rate constant *k*_*eff*_ with the PVP concentration in the photosensitizing system did not change after addition of chitosan into the reaction mixture: CT addition to the created photosensitizing systems resulted in some decrease of the *k*_*eff*_ values. This finding may indicate that a part of the porphyrin in the forming system binds to PVP, while the other part binds with chitosan.

**Figure 2:**
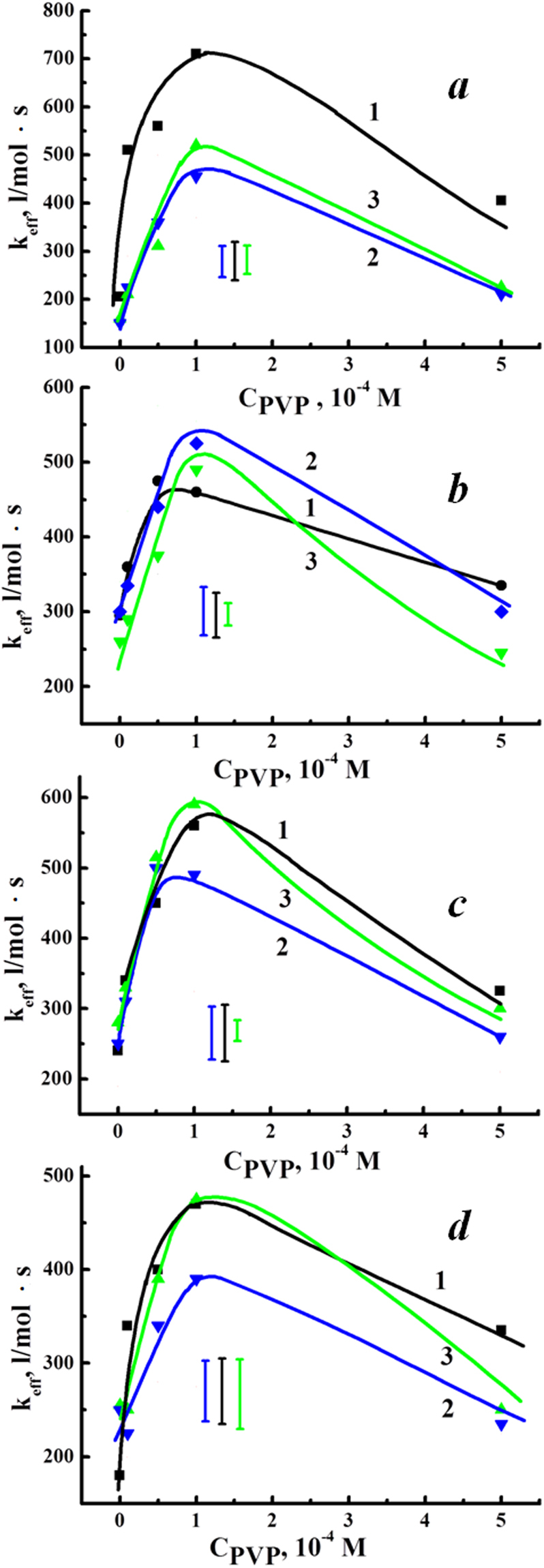
Dependencies of the effective rate constant *k*_*eff*_ of tryptophan (1 × 10^−4^ М) oxidation catalyzed by DMG–PVP–CT systems on the PVP concentration at different chitosan concentrations: ((**a**) −1 × 10^−6^ М, (**b)** −5 × 10^−6^ М, (**c**) −1 × 10^−5^ М, (**d**) −5 × 10^−5^ М) and different temperatures (Curves 1–24 °С, Curves 2–36.5 °С, Curves 3–39.0 °С). The DMG concentration is 5 × 10^−6^ М. The average errors for each plot are presented below the plots.

In Fig. 2, the dependencies of the rate constants *k*_*eff*_ of tryptophan oxidation on the PVP concentration are displayed for the ternary DMG-PVP-CT systems at different molar concentrations of chitosan and different temperatures: room temperature (24 °С), 36.5 °С and 39 °С. It should be noted that, in the case of only DMG in the aqueous system at a concentration of 5 × 10^−6^ М, the *k*_*eff*_ value at room temperature was ${k}_{eff}^{0}$ = 600 *l*/(*mol*·*s*).

As follows from Fig. 2, the dependencies of *k*_*eff*_ on the PVP concentration in the presence of the DMG-PVP-CT system have an extremum at all the studied ratios of PVP-CT. In the PVP concentration range of 5 × 10^−5^ М to 1 × 10^−4^ М and at the chitosan concentration of 1 × 10^−6^ М, the *k*_*eff*_ value at room temperature (Fig. 2а) exceeds ${k}_{eff}^{0}$ (by 1.2 times in the maximum). When the chitosan concentration is raised up to 5 × 10^−5^ М (Fig. 2d), the maximum *k*_*eff*_ value (at the temperature of 24 °С) and the minimum *k*_*eff*_ value (at 36.5 °С) are about 0.8 ${k}_{eff}^{0}$ and 0.6 ${k}_{eff}^{0}$, respectively. It is obvious that, for each characteristic temperature, a certain composition of the PPS-PVP-CT photosensitizing system may be selected, for which the *k*_*eff*_ value would be the highest.

As the experience shows, introduction of chitosan into photosensitizing systems in the conditions of native experiments allows a significant reduction of the PPS concentration for a given therapeutic effect. The presented data demonstrate a principal possibility for the selection of optimal ternary systems’ compositions for performing PDT within certain temperature ranges. The latter circumstance should be emphasized, since the detected non-monotonous character of the studied concentration dependencies at different temperatures reflects complex temperature-dependent conformation rearrangements in the considered ternary system, occurring when the relative ratio of components changes.

One may believe that the therapeutic effects of PDT using the created ternary systems at a certain ratio of components must depend on the size of associates formed in aqueous PPS-PVP-CT systems and on the appearance of complex bonds between fragments of the mentioned components. To obtain such information, we applied dynamic light scattering and analysis of fluorescence spectra of the created PPS-polymer systems in the aqueous medium.

**Figure 3:**
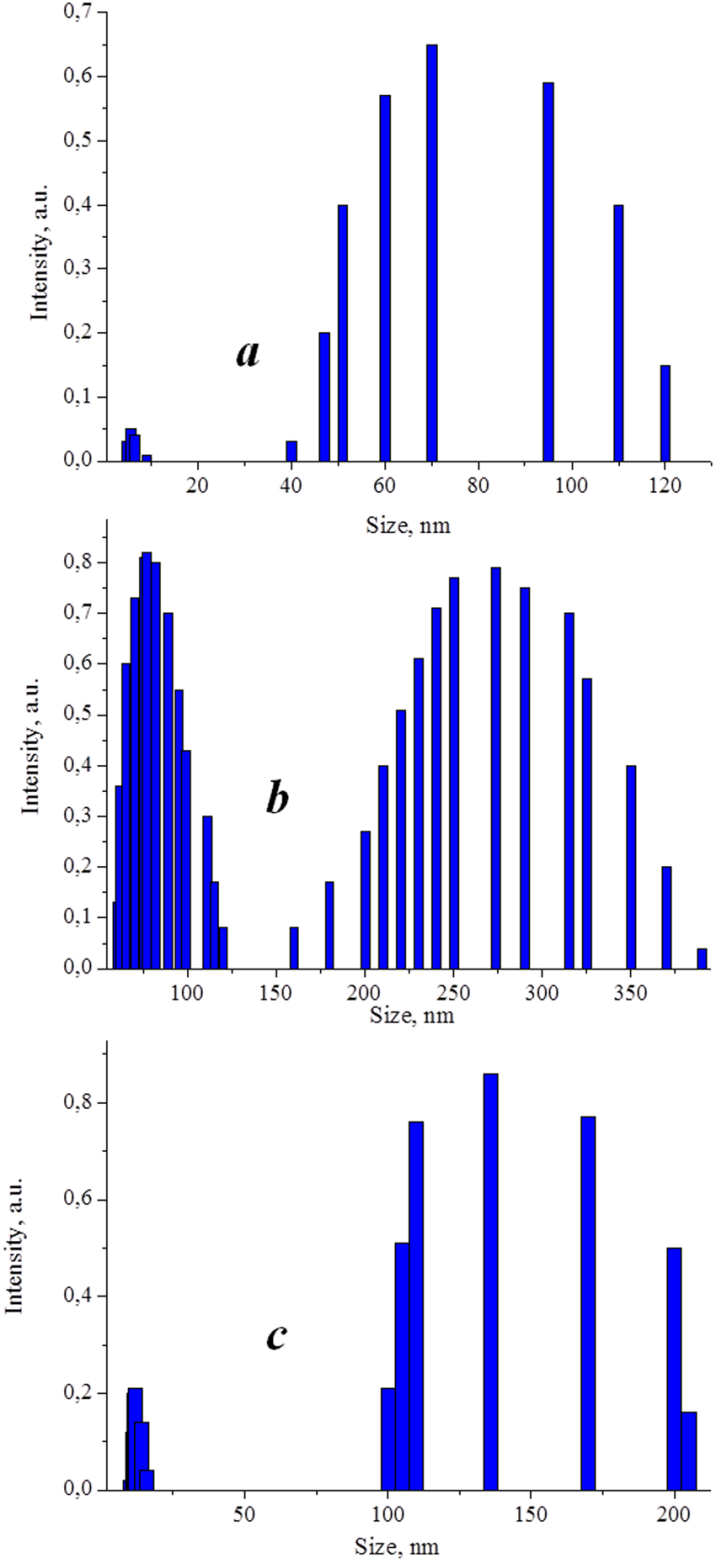
Size distribution of associates in aqueous solutions (**а**) DMG, (**b**) DMG-chitosan, (**c**) DMG-PVP-CT. [DMG] −5 × 10^−6^ М, [PVP] −1 × 10^−4^ М, [CT] −1 × 10^−6^ М.

Based on the analysis of the dynamic light scattering spectra, the data on the sizes of detected associates were gained. As follows from the presented data (Fig. 3a), the associates with the characteristic sizes of 90–130 nm (average size $\bar{d}$ ≈ 110 nm) are formed in an aqueous DMG solution (5 × 10^−6^ М). A small part of DMG exists in the form of particles with the sizes of 5–10 nm. In the presence of chitosan in the aqueous solution, the major part of DMG molecules bind to chitosan macromolecules, forming associates with the sizes of 150–370 nm, however, a part of DMG molecules remains unbound to chitosan, and a bimodal distribution of particles forms (Fig. 3b) with the characteristic sizes of 10–110 nm ($\bar{d}$ ≈ 60 nm) and 200–350 nm ($\bar{d}$ ≈ 270 nm). When adding PVP into the solution, the bimodality almost disappears (Fig. 3c): the associates with the characteristic sizes of 100–200 nm ($\bar{d}$ ≈ 150 nm) are mainly detected, that is apparently related to the ternary DMG-PVP-chitosan complexes. The detected changes in the size distributions indicate both the crucial role of polymer-polymer intermolecular interactions in the structuring of the ternary systems and a DMG participation in the corresponding structural rearrangements.

**Figure 4:**
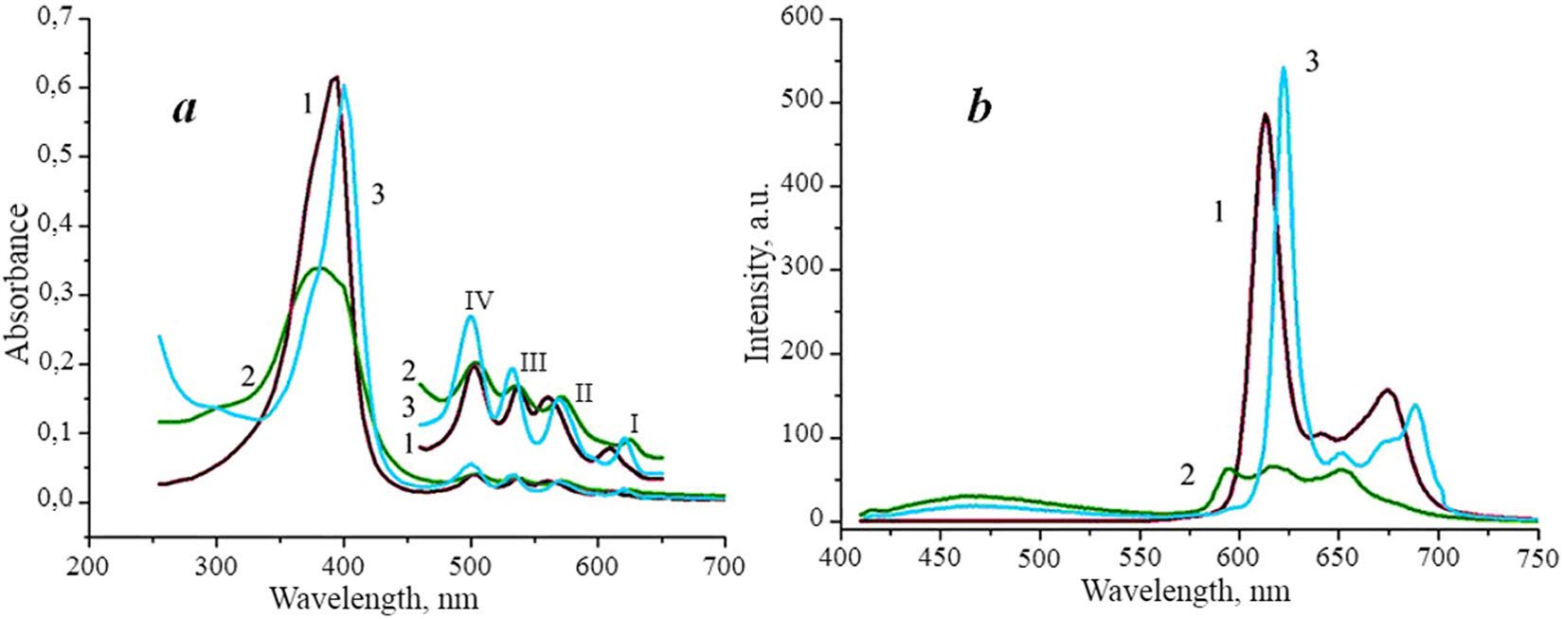
Absorption (**а**) and fluorescence (**b**) spectra of DMG^1^, DMG in the presence of chitosan^2^, ternary DMG-PVP-CT system^3^. [DMG] −5 × 10^−6^ М, [CT] −1 × 10^−6^ М, [PVP] −5 × 10^−4^ М. Excitation wavelength is 400 nm.

The changes in the absorption and fluorescence spectra excited at the wavelength of 400 nm (Fig. 4а,b) reflect the proceeding complex formation in the ternary DMG-PVP-CT systems. First of all, we observed a decrease in the intensity of all the absorption bands in the spectrum of the binary DMG-CT system, including those of the Soret band characteristic of DMG, which may be related to binding of DMG molecules to chitosan and their subsequent association. It is worth mentioning that such changes were observed at all the CT concentrations in the solution, even at the lowest one, 1 × 10^−6^ М. However, when PVP was added to the system, a certain growth in the intensity (by 10–20%) and “narrowing” of the Soret band took place, that may testify destruction of DMG associates and increase of the photosensitizing activity of such a system. The bathochromic shifts (by 5–10 nm) in the absorption spectra also indicate the processes of DMG associates’ disaggregation initiated by the presence of PVP in the system.

In this study, we did not set the goal of determining the reactive oxygen species generated after the absorption of UV radiation in the considered systems and the mechanisms of such generation. To illustrate the validity of our approach to the creation of ternary PPS-PVP-CT systems for efficient PDT therapy, we performed a study on the PDT treatment of model wounds of laboratory animals, in which we applied ternary complexes of polyvinylpyrrolidone and chitosan with photoditazine, a clinically approved photosensitizer for PDT. The studies were performed on animals (23 rats) divided into 6 groups, depending on the way of wound treatment in each group (Table 1).

**Table 1:** Distribution of animals by groups.

Group	*n*	Wound treatment
I	3	No treatment
II	4	Chitosan
III	4	Photoditazine, aqueous solution + irradiation
IV	4	Photoditazine –chitosan complex + irradiation
V	4	Photoditazine –PVP complex + irradiation
VI	4	Photoditazine –PVP–chitosan complex + irradiation

*n* – number of animals in the group.

The experimental Groups I–V served as a control for the basic experimental Group VI. In the study, we used a model of a full-thickness planar wound with Teflon restrictive rings described in^14^. The choice of the reagents’ concentrations (photoditazine and PVP) and the technique for the preparation of complexes, as well as the conditions of the wound modeling were similar to those in the mentioned study. The molar ratios of chitosan:polyvinylpyrrolidone (per unit) were 5:1. We used chitosan by Aldrich, with the medium molecular weight.

**Figure 5:**
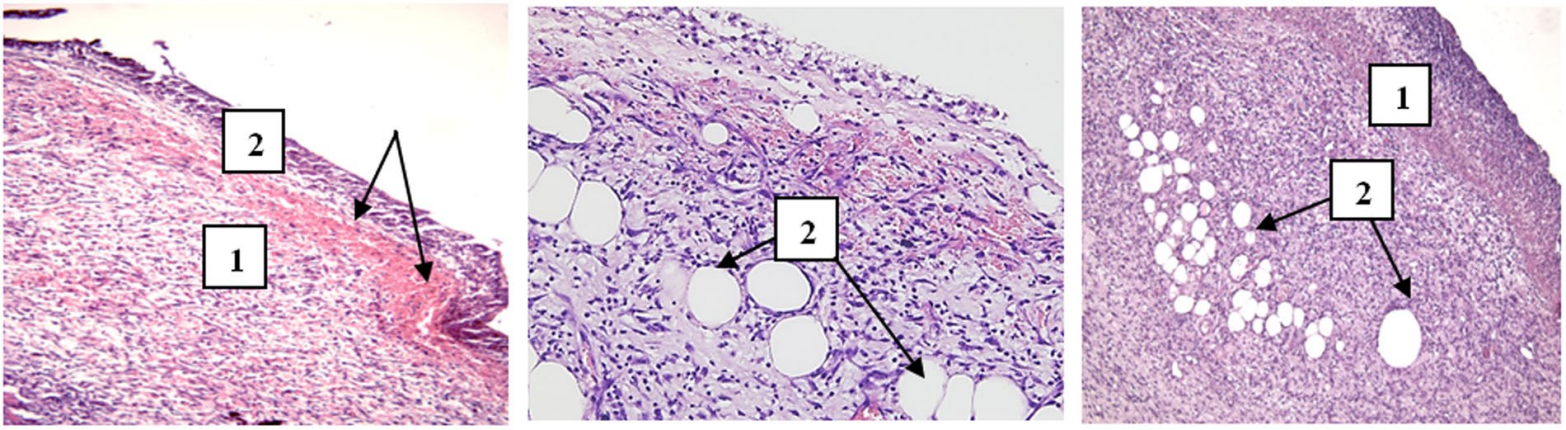
Fragments of the wound surface after PDT with the use of the photoditazine –chitosan complex (**a**), photoditazine – PVP complex (**b**), photoditazine – PVP-chitosan complex (**c**). (**a**) Group IV, day 4. Immature granulation tissue^1^, covered with fibrinogenous-leukocytic layer^2^. Arrows point to accumulations of erythrocytes between them. Hematoxylin-eosin stain, ×200. (**b**) Group V, day 4. 1 – Very thin fibrinogenous-leukocytic layer. Maturing granulation tissue with foci of diapedetic hemorrhages. 2 – Numerous mast cells with degranulation around the blood vessels. Hematoxylin-eosin stain, ×400. (**c**) Group VI, day 4. 1 – Continuous and thick layer of maturing granulation tissue. 2 – Fragments of fatty tissue. Hematoxylin-eosin stain, ×100.

It was shown that adding chitosan to the photoditazine solution (Group IV) almost did not decrease the hemorrhagic effect of PDT with the use of photoditazine which was discovered in^14^. Based on the external clinical signs and results of the histological studies, it was shown that PDT with the use of photoditazine-PVP-chitosan compositions (Group VI) had a notable anti-inflammatory and pro-regenerative effect. The introduction of chitosan into the photoditazine –PVP complex (Group VI) reduced the histotoxicity of photoditazine, as compared to the binary photoditazine-PVP system (Group V), that was testified by the absence of the hemorrhagic reaction in the wounds of the experimental Group VI. The application of PDT with the use of photoditazine complexes with PVP and chitosan enhanced the antibacterial effect to a greater effect than the photoditazine-chitosan complex (Group IV), since no bacterial cells were found in the wounds of animals in Group VI. At the same time, we detected bacterial cells in the wounds in Groups I-V. The ternary photoditazine-PVP-chitosan complex was shown to reduce the activity of inflammatory processes and enhance proliferation of fibroblasts, neoangiogenesis, growth and maturation of granulation tissue to a greater degree as compared to the photoditazine-PVP complex. Indeed, the granulation tissue thickness in the wounds of Group VI was 350 ± 50 µm at the fourth day of observation, while it was 206 ± 50 µm in Group V, and in Group IV the thickness of immature granulation tissue was (160–180) ± 50 µm (Fig. 5a–c).

## Methods

### Reagents

A water-soluble disodium salt of 3,8-di(1-methoxyethyl)deuteroporphyrin IX (dimegin, DMG^24^, synthesized by G.V. Ponomarev at the Institute of biomedical chemistry, Moscow) was used as a photosensitizer (Fig. 1а).

As an amphiphilic polymer, we used polyvinylpyrrolidone (PVP, M.W. 4 × 10^4^ Da, Sigma-Aldrich (Fig. 1b)). Chitosan (CT, Fig. 1c) with M.W. 2 × 10^4^ Da (Fig. 5с) was used as a biologically active polysaccharide, tryptophan (Sigma-Aldrich) was used as a substrate.

### Kinetics of tryptophan photooxidation

The reaction of tryptophan photooxidation in the aqueous medium in the presence of DMG and its complexes with polymers was conducted as follows. An aqueous solution of DMG (5 × 10^−6^ М) and the polymers (PVP and CT) was added to an aqueous solution of tryptophan (1 × 10^−4^ М). The concentration of the polymers usually varied between 1 × 10^−5^ and 5 × 10^−4^ М. Then the thermostated aqueous solution was stirred for 5 minutes, and the time count was started under the reaction mix illumination with the light of the wavelength λ = 400 nm (an AFS light-diode apparatus by Polironik, Moscow, with the power of 210 mW). The kinetics of the process was followed via the tryptophan concentration, which was measured by the decrease in the absorbance of the tryptophan UV absorption band (λ = 280 nm), while tryptophan was oxidized with the formation of peroxide products^25^. UV-Vis spectra of solutions were acquired with a Cary50 spectrophotometer (Varian, Austria).

For the comparative estimation of the activity of porphyrin-containing systems in the test reaction of tryptophan photooxidation in the aqueous medium, we introduced an effective specific rate constant *k*_*eff*_ calculated from the initial linear part of the kinetic plot *C*_*T*_ = *C*_*T*_(*t*) as:

1 $${k}_{eff}=(1/t)\cdot \,\mathrm{ln}({C}_{0T}/{C}_{T})/{C}_{PPS},$$

where *С*_0*T*_ is the initial substrate (tryptophan) concentration, *C*_*T*_(*t*) is the substrate concentration at the time point *t* (sec) of photooxidation, *С*_*PPS*_ is the concentration of a porphyrin photosensitizer. The error of *k*_*eff*_ measurements was 10%.

### Dynamic light scattering

The size of associates formed in the PPS-PVP-CT system was measured by dynamic light scattering with a Photocor (USA) laser light scattering goniometer, equipped by a He-Ne laser (10 mW, 633 nm). Autocorrelation functions of fluctuations of the scattered light intensity were measured using a 288-channel Photocor-SP correlator under a 90° scattering angle. The mathematical processing of the autocorrelation functions for the distribution of scattering particles by sizes was performed using the DynaLS software.

### Animal studies

All the invasive procedures in the study involving animals were performed in accordance with the international laws on the legal and ethical norms of animal studies, with the approval of the Ethical Committee of the Sechenov University (Statement No. 01-19 of 15.02.2017).

We used the same study design as in^14^, using a skin excision wound model with prevented contraction. The clinical monitoring and histological examination of the wounds were performed as described in^14^.

### Data availability

No datasets were generated or analysed during the current study.
