# Author Correction: Optimization of Photosensitized Tryptophan Oxidation in the Presence of Dimegin-Polyvinylpyrrolidone-Chitosan Systems

**DOI:** 10.1038/s41598-018-37181-7

**Published:** 2018-12-20

**Authors:** Anna B. Solovieva, Valeria V. Kardumian, Nadezhda A. Aksenova, Lyudmila V. Belovolova, Mikhail V. Glushkov, Evgeny A. Bezrukov, Roman B. Sukhanov, Svetlana L. Kotova, Peter S. Timashev

**Affiliations:** 1 N.N. Semenov Institute of Chemical Physics, Department of Polymers and Composites, 4 Kosygin St., 119991 Moscow, Russia; 2 Institute for Regenerative Medicine, I. M. Sechenov First Moscow State Medical University, 8 Trubetskaya St., Moscow, 119991 Russia; 3 A.M. Prokhorov Institute of General Physics, 38 Vavilov St., 119991 Moscow, Russia; 4 Department of Urology, I. M. Sechenov First Moscow State Medical University, 8 Trubetskaya St., Moscow, 119991 Russia; 5 Institute of Photonic Technologies, Research center “Crystallography and Photonics”, 2 Pionerskaya St., Troitsk, Moscow, 142190 Russia

Correction to: *Scientific Reports* 10.1038/s41598-018-26458-6, published online 23 May 2018

The Acknowledgements section in this Article is incorrect.

“This study has been supported in part by the Russian Science Foundation (Grant No. 16-13-10295) and by the grant from Sechenov First Moscow State Medical University, Russia, including preparation of the ternary DMG-PVP-CT compositions, analysis of their photocatalytic activity and temperature dependencies, acquisition of spectra, and in part by the Russian Foundation for Basic Research (Grants No. 17-02-00294, 16-32-00722), including measurements of the sizes of porphyrin aggregates and polymers by dynamic light scattering and laser elastic scattering. The authors wish to thank Dr. T.G.Rudenko and Dr. A.B.Shekhter for the animal studies.”

should read:

“This study has been supported by the Russian Science Foundation (Grant No. 16-13-10295) including preparation of the ternary DMG-PVP-CT compositions, analysis of their photocatalytic activity and temperature dependencies, acquisition of spectra and in part by the Russian Foundation for Basic Research (Grant No. 17-02-00294), including measurements of the sizes of porphyrin aggregates and polymers by dynamic light scattering and laser elastic scattering. The authors wish to thank Dr. T.G.Rudenko and Dr. A.B.Shekhter for the animal studies.”

